# The role of vascular mimicry as a biomarker in malignant melanoma: a systematic review and meta-analysis

**DOI:** 10.1186/s12885-019-6350-5

**Published:** 2019-11-21

**Authors:** Zhenhua Zhang, Saber Imani, Marzieh Dehghan Shasaltaneh, Hossein Hosseinifard, Linglin Zou, Yu Fan, Qinglian Wen

**Affiliations:** 1 Department of Oncology, The Affiliated Hospital of Southwest Medical University, Luzhou City, Sichuan Province 646000 People’s Republic of China; 2 Department of Biology, Faculty of Science, University of Zanjan, Zanjan, Iran; 3 Research Center for Evidence Based Medicine (RCEBM), Tabriz University of Medical Sciences, Tabriz, Iran

**Keywords:** Vasculogenic mimicry, Malignant melanoma, Diagnosis, Prognosis, Meta-analysis

## Abstract

**Background:**

Vasculogenic mimicry (VM) a microvascular system consisting of non-endothelial cells that is newly formed by aggressive tumors, has been proposed as an important therapeutic target in malignant melanoma (MM). We performed a systematic literature review to evaluate the diagnostic and prognostic accuracy of VM status for overall survival of MM patients.

**Methods:**

The quality of the included studies was evaluated using the QUADAS-2 tool. Diagnostic capacity of VM variables, including sensitivity, specificity, positive likelihood ratio (PLR), negative likelihood ratio (NLR), diagnostic odds ratio (DOR), and the area under summary receiver operating characteristic (SROC), were pooled using Meta-DiSc software.

**Results:**

A retrospective observational study was conducted based on twelve clinical studies including 978 clinically confirmed melanoma patients with proportion (P). VM+ melanoma cells were associated with poor prognosis in 38% of MM group (*P* = 0.35, 95% confidence intervals (CI): 0.27–0.42, *p < 0.001*). The pooled sensitivity and specificity were 0.82 (95% CI: 0.79–0.84) and 0.69 (95% CI: 0.66–0.71), respectively. Furthermore, the pooled PLR, NLR, and DOR were 2.56 (95% CI: 1.94–3.93), 0.17 (95% CI: 0.07–0.42), and 17.75 (95% CI: 5.30–59.44), respectively. Furthermore, the AUC of SROC was 0.63, indicating high reliability of VM status as a biomarker. Importantly, subgroup results suggested that VM+ status is a significantly accurate prognostic biomarker when diagnosed by the CD31−/PAS+ staining methods in Asian MM samples (*p < 0.001*).

**Conclusions:**

Our findings support the potential of VM status of tumors as a promising prognostic biomarker and emphasize an effective adjuvant therapeutic strategy in the prognosis of Asian MM patients.

## Background

Malignant melanoma (MM) is the most aggressive skin cancer and the most common skin disorder in Caucasians characterized by aggressive and progressive disease states, leading to major cancer-related morbidity and mortality, with an estimated global incidence of about 200,000 new cases per year and 50,000 cancer-related deaths in 2018 [1–3]. The incidence of MM has been rapidly increasing over the last 10 years in the Asian and Mediterranean population and in Singapore is diagnosed as the seventh and the eighth most common cancer among men and women, respectively [3–6]. Nevertheless, the Asian population has a notably lower risk of MM than Caucasians due to ethnic differences, anatomic distribution, histologic subtypes, and stage at diagnosis [6, 7]. Interestingly, Ultraviolet (UV) radiation, race, lifestyle, and genetic differences are the most important reasons for the high mortality rate of melanoma which can be decreased via early-stage detection and prevention [8–13]. Dermoscopy and intrinsic molecular subtyping of melanoma have been widely accepted as accurate diagnostic methods with more than 50% accuracy compared with the clinical diagnosis in patients with MM [14, 15].

Recent investigations identified a new non-angiogenesis-dependent pathway entitled vasculogenic mimicry (VM), which refers to a vessel-like structure formed by extremely aggressive tumor cells that imitate endothelial cells [16, 17]. VM has been considered as a cancer hallmark that can independently facilitate tumor neovascularization by the formation of fluid-conducting and vascular endothelial cells [18–20]. VM could dedifferentiate into numerous cellular phenotypes and obtain endothelial-like features, resulting in the formation of the de novo matrix-rich vascular-like network, such as plasma and red blood cells [21, 22]. The co-generation of endothelial cells, channels, laminar structures, and heparin sulfate proteoglycans are the main pathophysiological characteristics of VM in human melanoma patients [23–25]. Aggressive VM+ tumor cells are characterized by a higher expression of the basement membrane extracellular matrix (ECM) components laminin5γ2 and metalloproteinases (MMPs)-1, − 2, − 9, and − 14 [21, 22, 26]. In highly aggressive melanoma cells downregulation of vascular endothelial cadherin and upregulation of ECM components promotes the perfusion of the VM pathway [19, 21]. Ultimately, the VM+ melanoma cells are associated with more aggressive and metastatic tumor biology.

Accumulating evidence suggests that VM is associated with poor prognosis in various malignant human tumors, including breast [27], colorectal [28], prostate [29], hepatocellular carcinoma [30], lung [29], ovarian [31], gastric [32], and bladder cancers [33]. Despite numerous experimental studies, the prognostic value of VM status for survival in MM patients is still controversial and inconclusive. Understanding the role of VM in MM pathogenesis may help to develop effective treatments for tumor invasion and to overcome drug resistance in MM [34].

Hence, we conducted a quantitative systematic review along with a comprehensive meta-analysis investigation based on eligible studies to resolve inconsistent and often ambiguous findings. Furthermore, we identified the prognostic accuracy of VM status in cancer patients to predict other clinical pathological feature outcomes of MM.

## Methods

This systematic review and meta-analysis was performed according to the recommendations of the Preferred Reporting Items for Systematic Reviews and Meta-Analyses (PRISMA) statement guidelines [35].

### Search strategy and study selection

MEDLINE electronic databases of Pubmed, Embase, Wiley Online Library, Web of Science, Science Direct, Cochrane library, and VIP-Google Scholar were searched to assess the prognostic value of VM in melanoma patients prior to April 18, 2019. Different spelling and synonyms were combined applying Boolean “OR” and main terms were linked applying Boolean “AND” to identify all relevant studies. The search string was conducted using MeSH terms and following main headline terms or free words based on the research question (both the UK and US spellings), such as: “vascular mimicry OR vasculogenic mimicry OR tumor cell-lined vessels OR tumor-derived endothelial cells” AND “prognosis OR survival OR outcome” AND “melanoma OR basal cell carcinoma OR squamous cell carcinoma OR cancer OR neoplasms OR malignant melanoma OR basal-cell skin OR squamous-cell skin OR skin”. The comprehensive literature search strategies are detailed in Additional file 1: Table S1, which were separately retrieved and screened by four researchers (ZZ, SI, HH, and MDS).

### Inclusion/exclusion criteria

The current meta-analysis covered all prospective and randomized controlled trials (RCTs) that were considered eligible if they met the following criteria: (*i*) Melanoma patients were confirmed by immunohistochemical or histochemical tests. (*ii*). VM+ tumor tissue samples were assessed by classical staining of the specimens, including positive Periodic Acid-Schiff (PAS) and/or negative endothelial cell markers, CD34 or CD31; (*iii*) No previous systemic treatment for metastatic disease. Likewise, we excluded all non-comparative, review, case-control, conference abstracts, meeting reports, commentaries, and unrelated articles, as well as family-based, in vitro, and animal studies. Moreover, we excluded duplicate studies, continued work of previous publications, and poor quality studies, as well as those with incomplete and/or missing data such as sample size and VM frequency.

### Data extraction and quality assessment

All selected articles were reviewed independently by three researchers (ZZ, SI, and MDS) according to the population, intervention, control, and outcomes (PICO) principle [36] and any disagreements or inconsistencies in a search process were addressed through consultations and debate. If an acceptable consensus was not reached, a fourth partner (QW) would resolve these disagreements based on the original data. The key demographics and clinicopathological information of all qualified data collections were summarized in Tables 1 and 2. These included the first author’s name, publication year, total cases, gender, country of origin population, age, follow up time, VM+ or VM- rate, analyzing methods of VM, Clark level, and location of sampling. In addition, we emailed corresponding authors to obtain any additional or missing information, as well as original data needed for the meta-analysis. If the above data were not cited in the original study or no reply was received by email, the item was reported as “not reported (NR)”. All eligible studies were assessed based on the Newcastle-Ottawa scale (NOS) [37] and Quality Assessment of Diagnostic Accuracy Studies 2 (QUADAS-2) [38] protocols. In addition, the probability of bias was calculated based on the criteria from the Cochrane Collaboration’s tool (Cochrane handbook for systematic reviews of interventions version 5.1.0.).

**Table 1:** Demographic information of included studies

First author (Ref.)	Year	SS	Gender (M/F)	Population (ethnicity)	Age		Flow time (months)	NOS^a^
					≤ 30	> 30		
Maniotis AJ, [34]	1999	234	NA	USA (C)	NA	NA	480	7
Massi D, [35]	2004	45	22/23	Italy (C)	–	45	120	8
Hillen F, [37]	2008	58	18/40	Netherland (C)	16	42	120	8
Zhang SH, [38]	2009	124	67/57	China (A)	NA	NA	250	6
Shi L, [39]	2010	45	31/14	China (A)	–	45	100	7
Beurden BV, [40]	2012	123	58/65	Netherland (C)	41	82	200	9
Itzhaki O, [41]	2013	15	10/5	Israel (C)	1	14	NA	7
Song H, [42]	2015	62	34/28	China (A)	NA	NA	90	7
Baocun S, [43]	2015	60	NA	China (A)	NA	NA	39	8
Zhao X, [44]	2015	79	47/32	China (A)	NA	NA	200	7
Liang X, [45]	2017	81	54/27	China (A)	59	22	100	7
Zhang W, [46]	2017	52	36/16	China (A)	37	15	80	7

*Abbreviations*: *Ref.* Reference, *SS* Sample size, *M* Male, *F* Female, *C* Caucasian, *A* Asian, *NOS* Newcastle-Ottawa scale, *NA* Not avalibale

^a^The quality of non-randomized studies will be appraised using the Newcastle-Ottawa scale (NOS), categorized into three groups: the selection of the study groups; the comparability of the groups; as well as the ascertainment of either the exposure or outcome of interest for case-control or cohort studies respectively

**Table 2:** Main clinicopathological and vasculogenic mimicry characteristics of all relevant studies

First author (Ref.)	VM state n (%)		Methods of VM assay	Clark level^a^			Location		
	VM+	VM-		ІІІ	ІV	V	Head and neck	Trunk	Extremities
Maniotis AJ, [34]	106 (45)	128 (55)	PAS	–	234	–	234	–	–
Massi D, [35]	15 (30)	30 (70)	CD31/PAS	–	45	–	6	21	18
Hillen F, [37]	22 (38)	36 (78)	PAS	12	22	24	6	17	22
Zhang SH, [38]	54 (43)	70 (57)	PAS	–	65	59	16	46	62
Shi L, [39]	11 (27)	34 (73)	CD31/PAS	33	12	–	32	13	–
Beurden BV, [40]	42 (34)	81 (66)	PAS	23	51	49	–	112	11
Itzhaki O, [41]	14 (93)	1 (7)	CD31/PAS	2	8	5	2	10	3
Song H, [42]	25 (41)	37 (59)	CD31/PAS	14	45	3	62	–	–
Baocun S, [43]	10 (5)	50 (95)	CD31/PAS	–	38	22	NA	NA	NA
Zhao X, [44]	36 (45)	43 (55)	CD34/PAS	46	33	–	21	43	15
Liang X, [45]	35 (43)	46 (57)	CD34/PAS	–	41	40	34	28	19
Zhang W, [46]	106 (45)	128 (55)	CD34/PAS	–	22	30	22	16	14

*Abbreviations*: *Ref.* Reference, *VM* Vasculogenic mimicry, *PAS* Periodic acid schiff’s, *NA* Not available

^a^All tissue samples are formalin-fixed, paraffin-embedded and categorized by five anatomical levels of Clark’s staging system

### Statistical analysis

The current systematic meta-analysis was carried out applying Comprehensive Meta-Analysis (CMA) software (Biostat, Englewood, NJ 07631, USA, version 2.2.064). The diagnostic accuracy and ROC curves were conducted on MetaDiSc (version 1.4). Additionally, the quality of study was calculated by RevMan version 5.2 [39, 40]. Pooled specificity, pooled sensitivity, negative likelihood ratio (NLR), positive likelihood ratio (PLR), and diagnostic odds ratio (DOR) were calculated with corresponding 95% CIs to evaluate the diagnostic value of VM. Furthermore, the summary receiver operating characteristic (SROC) curve was calculated for the involved studies with an overall area under the curve (AUC). Results of the meta-analysis were reported as a proportion (P) with 95% confidence intervals (CIs). All data were reported as mean ± standard deviation (SD) or as median (range). As well, a description of qualitative variables as number and percentage are given. The chi square-based Q-test was applied to testify between-study heterogeneity. Subgroup analysis was performed to identify the source of existing heterogeneity between the VM+ and available sub analyses such as sample size, race, and VM detection methods. Publication bias was assessed using Begg’s funnel plots [43] and Egger’s regression test [44]. A value of “Pr > |z|” less than 0.05 was considered to be a potential publication bias. All reported *p* values were two-sided and *p* < 0.05 was considered statistically significant.

## Results

### Description of studies

**Fig. 1:**
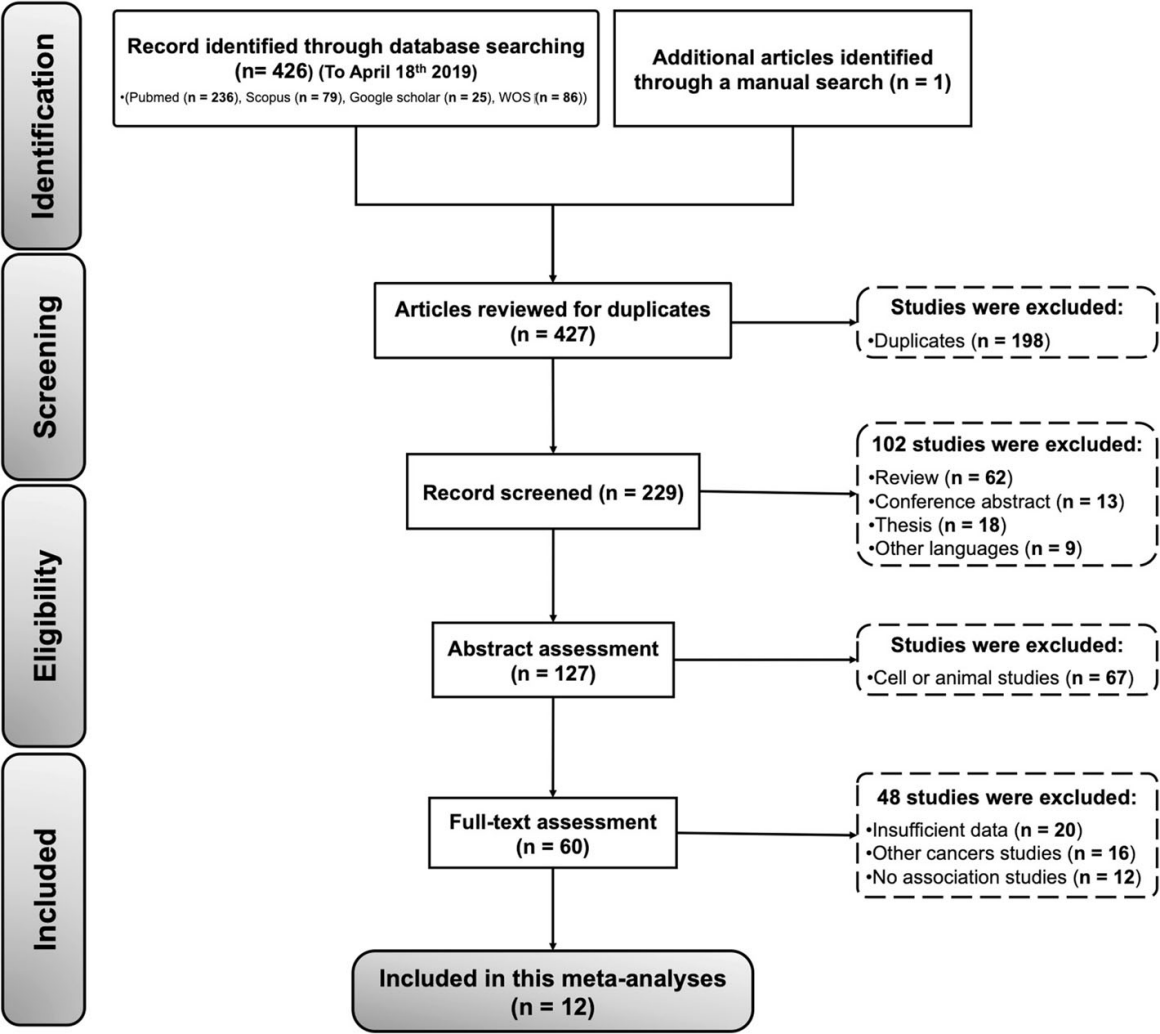
Flow diagram of included studies (following PRISMA guidelines, n = number of studies)

A detailed PRISMA flowchart of the study identification, screening, and exclusion process is shown in Fig. 1. The primary manual search yielded 426 potentially eligible publications through searching of electronic databases and 1 record. After excluding duplicate studies (198 studies), 229 publications were kept for screening, of which 102 records were excluded according to the inclusion and exclusion criteria for database searching. Then, the remaining 127 articles were further assessed by abstract reviewing, and 67 studies were discarded being either cell or animal studies. Following careful review of titles and abstracts, full-text articles of 60 studies were assessed for suitability. Twenty studies were excluded for obvious irrelevance, 16 studies were precluded for dealing with other types of cancer, and 12 studies were dismissed due to having no related assays. Finally, 12 studies were retained in this meta-analysis [42, 45–54].

### Characteristics of studies

The demographic information of all relevant studies is detailed in Table 1. According to this table, a total of 12 studies with 978 MM patients dating between 1999 and 2017 were included in this systematic review and meta-analysis. Seven studies were conducted in people of the Asian race (58.4%) [41, 42, 49, 50, 52–54], four studies (33.4%) in European countries [45, 48, 51, 55], one study in the USA (8.2%) [47], and no study from African populations. Gender subgroups among the 978 patients included 377 male and 307 female patients. The major clinicopathological features of the included studies are shown in Table 2. More than 80% of the MM patients were diagnosed by histopathological tests. PAS staining combined with endothelial markers (CD31 or CD34) is a commonly used method for identification of tumor VM in paraffin-embedded tissue specimens and was done in 8 studies (66.7%) [41, 42, 45, 50, 52–55], as well as PAS staining in 4 studies [47–49, 51]. Moreover, significant predictors of VM+ in both adjusted and unadjusted analyses were Clark level IV or V (84.4%). Finally, 11 studies reported the association between VM and clinicopathological parameters regarding OS [41, 42, 45–54], with the follow-up period ranging from 39 to 480 months.

### Quality assessment

All 12 papers were methodologically assessed according to NOS and QUADAS-2 quality evaluation standards of the Cochrane Reviewer handbook. Both systems’ tools focused on the study dependent on the methodology. Overall, the average NOS score was approximately 7.4 out of 12, which could be classified nearly in the high quality group. For each study, the NOS score is given in Table 1. Furthermore, QUADAS-2 results confirmed that no significant bias was detected in the present meta-analysis. Details of the quality evaluation of eligible studies according to the NOS score are summarized in the Additional file 1: Table S3. The reviewers’ decisions about each risk of bias and applicability concerns graph are presented as percentages across selected studies. Additional file 2: Figure S1 shows all parameters of QUADAS-2 assessment individually. In this study, no significant bias and applicability concerns were found in any of the selected studies.

### Outcome of the meta-analysis

**Fig. 2:**
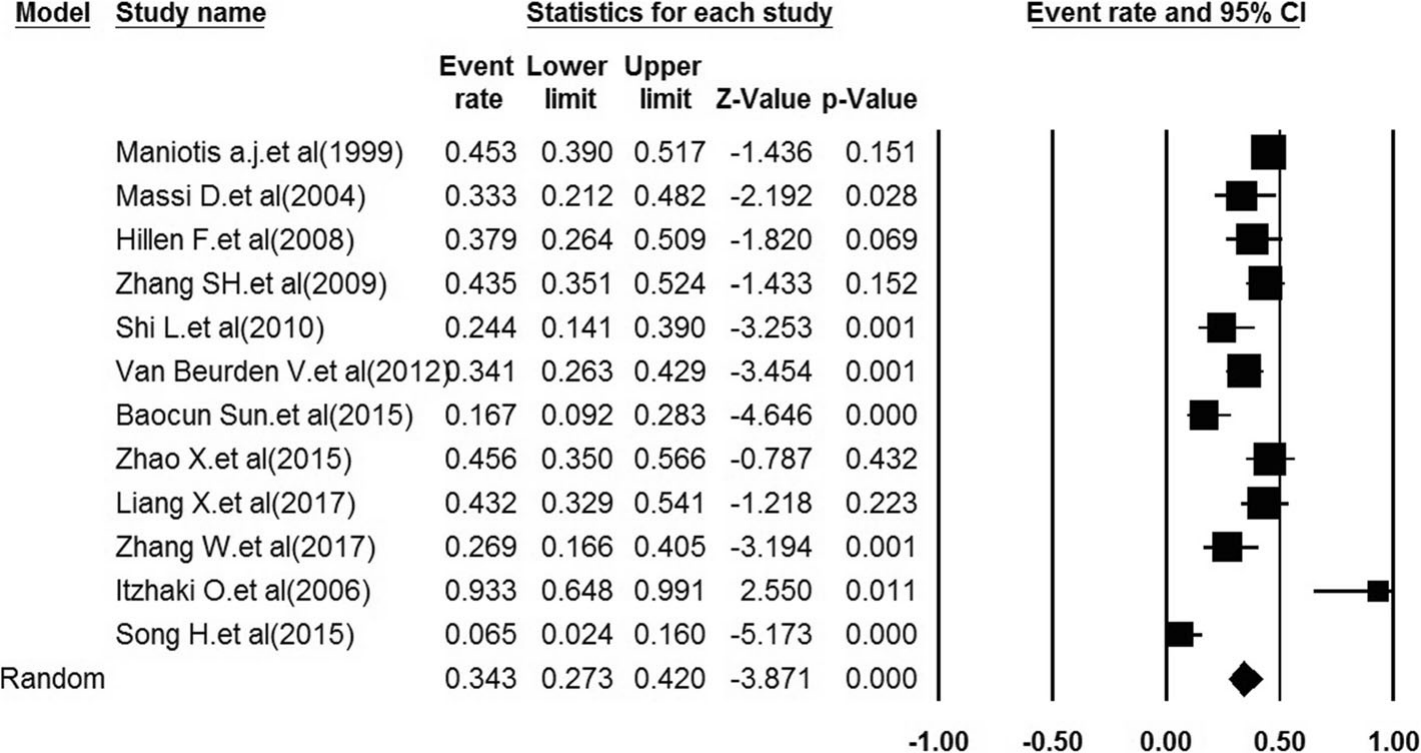
Forest plot of proportion ratios (P) in the random effect model. These plots show the prognostics accuracy for all objective response analyses

The relationship between VM+ and overall survival of MM patients was identified applying the pooled proportions test method. We used a random effect approach because the heterogeneity of the overall prognosis was relatively high, which is shown across the study (I_2_ = 79.8, *p-value* < 0.001). Based on heterogeneous cross of 12 studies, VM was associated with poor prognosis in 38% of MM group compared to the VM-group (*P* = 0.35, 95% confidence intervals (95% CIs): 0.27–0.42, *p-value < 0.001*). Therefore, these results suggested that VM+ indicated a poorer prognosis for MM patients (Fig. 2).

### Diagnostic accuracy

**Fig. 3:**
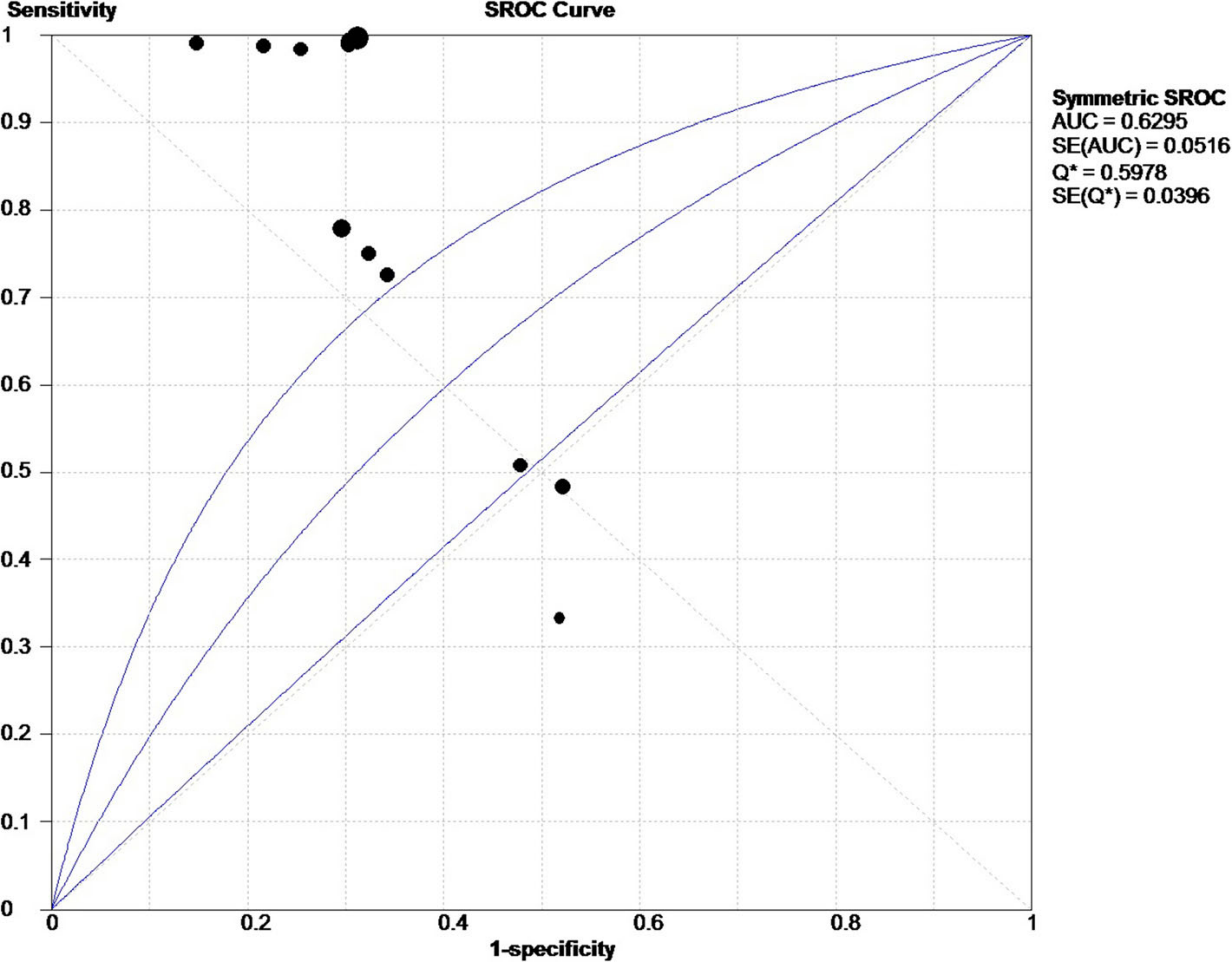
Summary receiver operating characteristic (SROC) curve for VM in the diagnosis of MM cancer

**Fig. 4:**
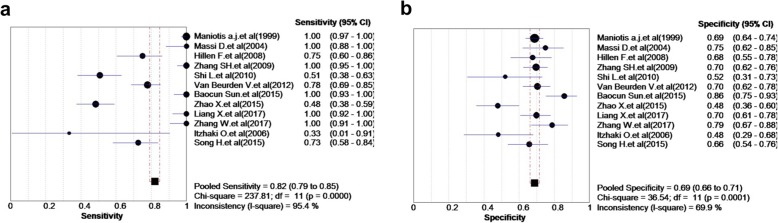
Forest plot of pooled sensitivity (**a**) and specificity (**b**) for VM in the diagnosis of MM cancer

The effect of heterogeneity on the diagnostic threshold was evaluated based on the Spearman correlation coefficient. Figure 3 presents the forest plots of pooled sensitivity and specificity, with the 95% CIs for individual studies. According to the results, the overall pooled sensitivity of VM+ tumor was 0.82 (95% CI: 0.79–0.84, Fig. 4a), while the specificity of VM+ tumor was 0.69 (95% CI: 0.66–0.71; Fig. 4b), among the 12 included studies. Furthermore, the overall pooled results for PLR, NLR, and DOR were 2.56 (95% CI: 1.94–3.93), 0.17 (95% CI: 0.07–0.42), and 17.75 (95% CI: 5.30–59.44), respectively.

### Subgroup analysis

**Fig. 5:**
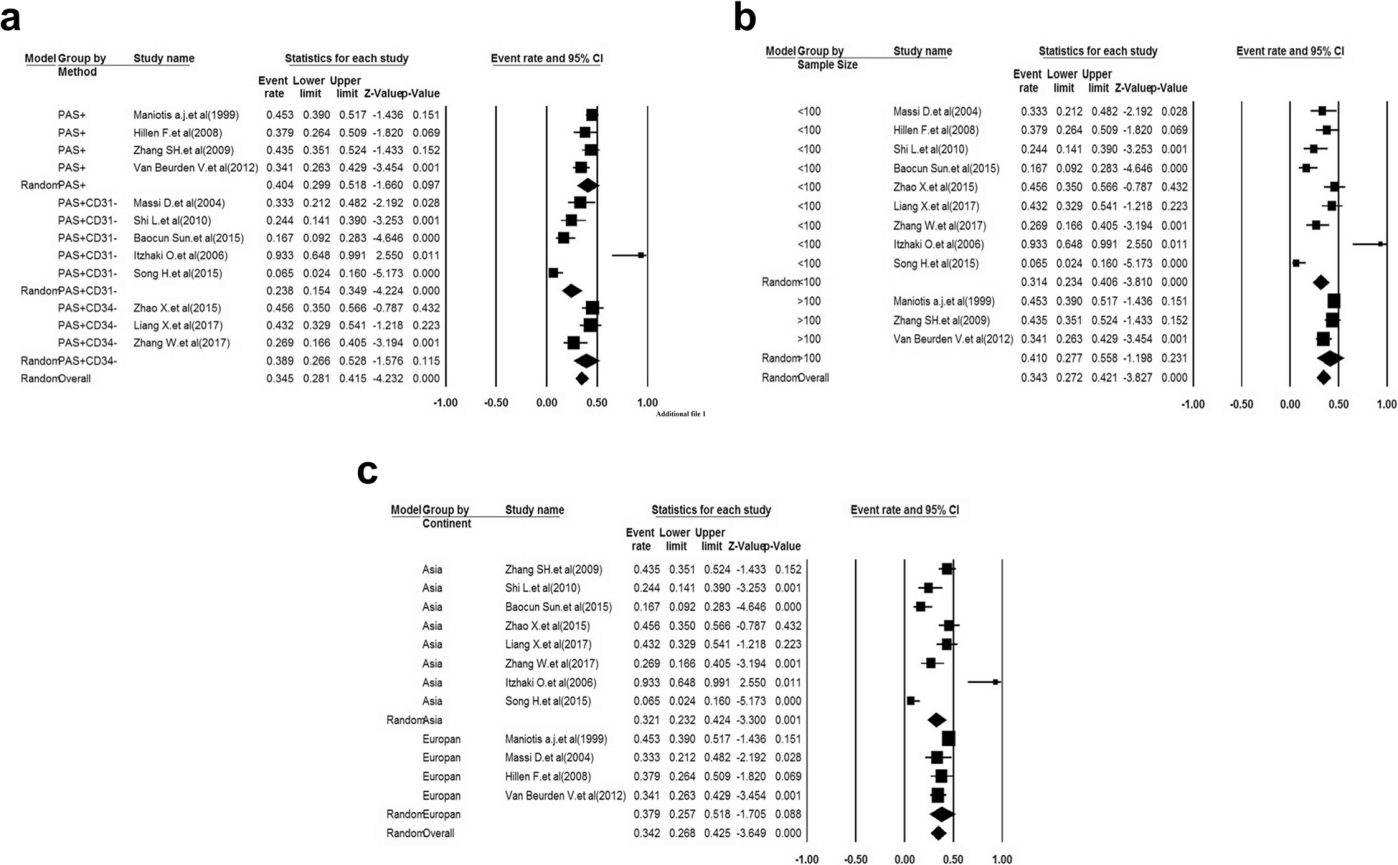
Funnel plot of the sub-analysis parameters. Forest plots showed that MM cancer was associated with detection methods of VM (**a**), sample size (**b**), and race (**c**). CIs, confidence intervals. Weights are from random effects analysis

Associations between VM+ and the possible demographic and clinicopathological features of MM patients are listed in Table 3. According to the results, none of the above covariates contributed to the heterogeneity (all *p > 0.05*). Therefore, according to those covariates, the pooled sensitivity, specificity, PLR, NLR, DOR, and AUC were measured for significant sub-analysis parameters. We detected statistically significant relationships between VM and sample size, VM and race, as well as between VM expression and staining method (Fig. 5). As shown in Fig. 5a and Table 3, VM+ is a potentially accurate prognostic biomarker in CD31−/PAS+ (*P* = 0.24, 95% CI: 0.15–0.35) compared to CD34−/PAS+ (*P* = 0.39, 95% CI: 0.27–0.42) and the PAS+ staining subgroups (*P* = 0.40, 95% CI: 0.30–0.52). As a result, the CD31−/PAS+ staining method is a relatively accurate diagnostic method for detection of the VM, with 75% sensitivity and 70% specificity. The subgroup analysis was performed based on sample size (≤100 vs. > 100; Fig. 5b). The proportion of the population with a large sample size (3 studies with more than 100 MM cases) was 0.41 (95% CI: 0.28–0.56; *p* = 0.12); while that of a sample size with less than 100 MM patients (9 studies) was 0.31 (95% CI: 0.23–0.41; *p < 0.001*). Meanwhile, the highest specificity, NLR, and AUC in sample sizes less than 100 suggested that VM is more accurate in diagnosis of smaller sample sizes. Interestingly, our results show that overexpression of the VM was a high-risk prognosis factor in Asian populations (7 studies with 503 cases; *P* = 0.32; 95% CI: 0.23–0.42; *p < 0.001*; Fig. 5c). As seen in Table 3 and Fig. 5c, the pooled sensitivity and specificity were higher in the Asian patients compared to the Caucasian patients (85% vs. 69 and 78% vs. 68%, respectively). On the other hand, we could not find any significant correlation between the VM+ melanoma samples and gender, age, Clark level, or location of sampling (Data not shown).

**Table 3:** Subgroup analyses of the included studies

Subgroup analyses	n (%)	P (95% CI)	I2 (%)	*P*-value	Sensitivity (95% CI)	Specificity (95% CI)	PLR (95% CI)	NLR (95% CI)	DOR (95% CI)	AUC
Sample size										
≤ 100	9 (75)	0.31 (0.23–0.41)	81.72	< 0.001	0.85 (0.82–0.88)	0.78 (0.60–0.75)	2.72 (1.98–3.75)	0.11 (0.32–0.40)	26.44 (5.41–129.09)	0.63
> 100	3 (25)	0.41 (0.28–0.56)	52.94	0.231	0.69 (0.60–0.76)	0.68 (0.66–0.71)	2.139 (1.07–4.25)	0.35 (0.089–1.37)	7.03 (0.917–53.46)	0.61
Ethnicity										
Asian	8 (66)	0.32 (0.23–0.42)	85.22	< 0.001	0.91 (0.87–0.93)	0.70 (0.66–0.72)	3.048 (2.681–3.46)	0.07 (0.015–0.34)	45.42 (7.63–270.56)	0.55
Caucasian	4 (34)	0.38 (0.26–0.52)	44.27	0.088	0.72 (0.67–0.77)	0.67 (0.62–0.71)	2.189 (1.23–3.88)	0.29 (0.095–0.89)	9.31 (1.73–50.1)	0.75
Detection methods										
PAS+	4 (34)	0.40 (0.30–0.52)	35.04	0.097	0.75 (0.69–0.81)	0.69 (0.65–0.72)	0.09 (0.018–0.47)	2.98 (2.61–3.41)	34.34 (5.12–230.22)	0.75
PAS + CD31-	5 (41)	0.24 (0.15–0.35)	84.55	< 0.001	0.9 (0.86–0.92)	0.70 (0.64–0.75)	2.36 (1.2–4.64)	0.29 (0.073–1.17)	9.24 (1.22–69.99)	0.60
PAS + CD34-	3 (25)	0.39 (0.27–0.42)	59.52	0.081	0.73 (0.66–0.79)	0.66 (0.59–0.72)	2.37 (0.92–6.14)	0.07 (0.01–37.37)	33.43 (0.12–92.24)	0.46
Total										
12 (100)		0.35 (0.27–0.42)	79.75	< 0.001	0.82 (0.79–0.84)	0.69 (0.66–0.71)	2.56 (1.94–3.93)	0.17 (0.065–0.42)	17.75 (5.301–59.44)	0.629

*Abbreviations*: *PAS* Periodic acid schiff’s, *n* Number, *P* Proportion, *95% CI* 95% confidence intervals, *PLR* Positive likelihood ratio, *NLR* Negative likelihood ratio, *DOR* Diagnostic odds ratio, *AUC* Area under the characteristic

### Publication bias and sensitivity analysis

**Fig. 6:**
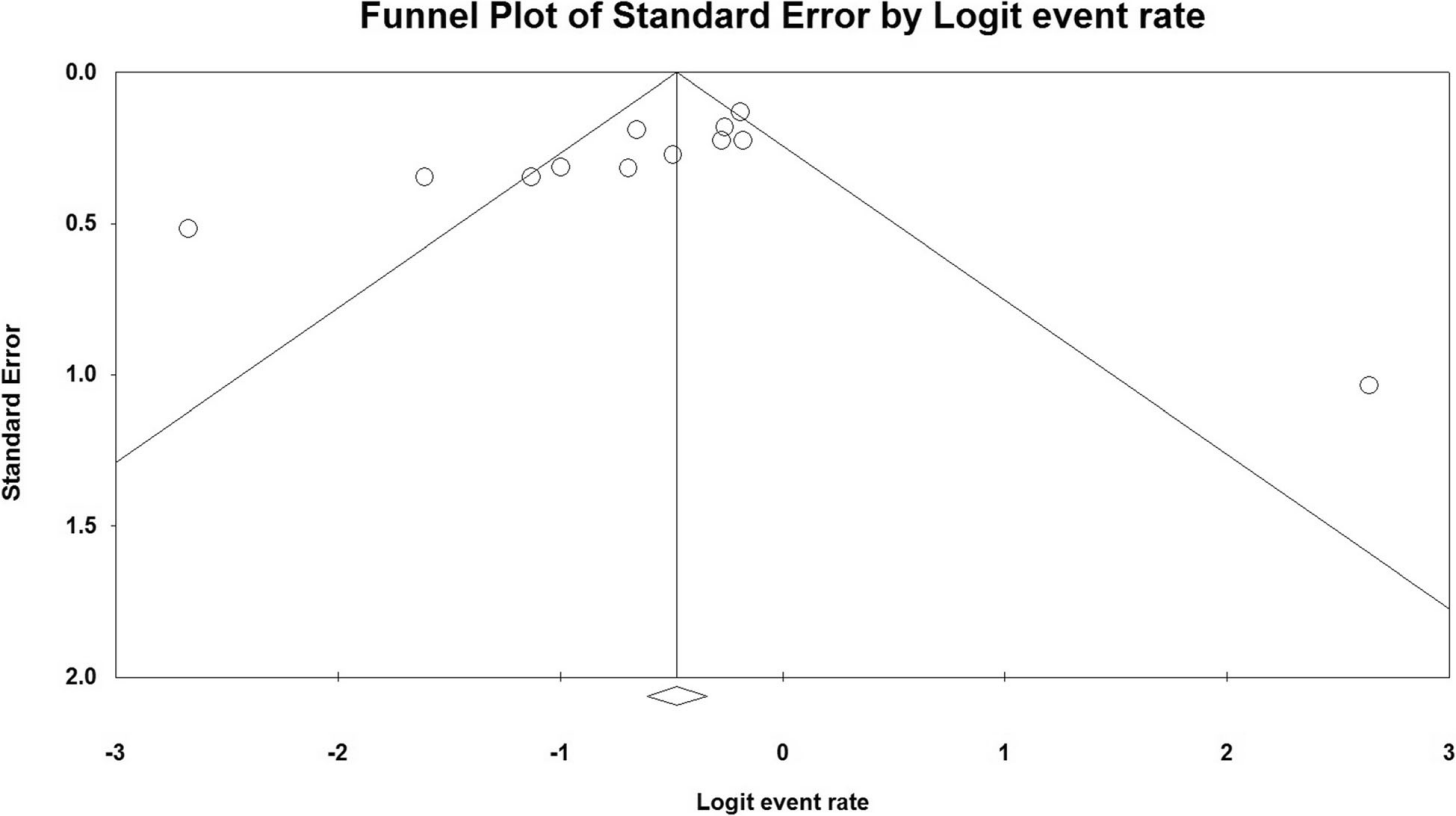
Funnel plots for the detection of a publication bias. All enrolled 12 studies represent by each point for the specified association, individually. The vertical and horizontal axes represent the standard error of a logarithmic proportion and the proportion limits, respectively

The publication bias and sensitivity were analyzed using Funnel plots and empirically utilizing regression tests according to Begg’s rank test. The analysis was conducted by excluding a single study at a time. A symmetric inverted funnel shape in this study implies a ‘well-behaved’ data set in which publication bias is improbable. Following exclusion of ten studies, there was no obvious statistical evidence for publication bias in our meta-analysis (t = 1.41; *p* = 0.19) (Fig. 6). Hence, the results of the current meta-analysis are credible and stable, with no noticeable publication bias influencing the overall results.

## Discussion

To the best of our knowledge, this is the first meta-analysis study to identify the prognostic value of VM status in advanced melanoma patients. Our results indicate that 38% of MM patients with VM+ have a poor prognosis (*P* = 0.35, 95% CI: 0.27–0.42, *p* < 0.001). Moreover, a significant association was identified in the pathologic features of the VM+ melanoma samples by race, sample size, and VM detection methods, which adversely influenced cancer survival. In the current study, the AUC of SROC was 0.63, indicating the high accuracy of VM status as a biomarker for MM. In addition, our pooled results provided convincing evidence for a significant positive relationship between VM and small sample size.

Accumulating evidence indicates that VM is a new model of tumor microcirculation in highly aggressive malignant tumor cells [16, 17]. Recently, in vivo and in vitro studies have shown that twist-related protein 1 (Twist1), neurogenic locus notch homolog protein 4 (Notch4), hypoxia inducible factor (HIF)-1a, EPH receptor A2 (EphA2), matrix metalloproteinase (MMP)-1, 2, − 9, − 14, and vascular endothelial (VE)-cadherin are potential therapeutic targets and prognostic indicators in VM+ tumor samples [22, 56]. Moreover, these studies suggested that VM+ tumor samples are resistant to common antiangiogenic drugs, such as apatinib, bevacizumab, and sunitinib [23, 34, 57]. The high ratio of neovascularization in VM+ tumors promotes angiogenesis, metastasis, and tumor growth along with extensive hypoxia and necrosis. It also induces recruitment of various pro-angiogenic factors, such as bone marrow-derived CD45^+^ myeloid cells, pericyte progenitor cells, and mature F4/80^+^ tumor-associated macrophages [58, 59]. Varying locations and heterogenic morphology of MM tumors display a close relationship with VM formation, which represents a noteworthy challenge for dermatologists [16, 60].

Our results clearly show that VM has a negative effect on the overall survival of MM patients with a risk ratio of 0.35 (95% CI: 0.27–0.42, *p < 0.001*). Furthermore, our findings from sub-analyses underlined the status of VM formation in MM patients. Our results reveal a strong association between VM+ and sample size, between VM+ and race, as well as between VM+ and detection method of VM (*p < 0.001*). Our findings suggest that VM status can be a significantly accurate prognostic biomarker when diagnosed by CD31−/PAS+ staining, with a relatively accurate diagnostic value for VM detection (75% sensitivity and 70% specificity). Also, the results of subgroup analyses implied a better diagnosis of VM in small sample sizes compared to that in samples containing greater than 100 cases (P: 0.31, 95% CI: 0.23–0.41; *p < 0.001*), with a pooled sensitivity of 85% and specificity of 78%. Interestingly, our results propose VM status as a more promising, accurate biomarker and target for MM diagnosis and therapeutics in Asian patients than in Caucasian patients, with a pooled sensitivity of 91% and specificity of 70.5% vs. Lifestyle factors such as UV radiation exposure and nutrition are synergistically contributing to the prevalence of MM [61, 62]. Compared to Caucasians, Asian MM patients are diagnosed at older ages; hence, in Asia we face a large population of old MM patients [4, 12]. But considering that our study was limited to a small sample size of cases in the Caucasian group (475 cases), more large-sized studies among the Caucasian MM population should be performed to obtain a comprehensive result [61]. It is known that VM+ tumor sample profiling is more accurate in the Asian population than in the Caucasian population [62]. The meta-analysis showed that the CD31−/PAS+ staining is a more accurate detection method for VM+ tumor samples than CD34−/PAS+ and PAS+ staining. Meanwhile, this meta-analysis suggests that postoperative detection with CD34- and/or CD31- of VM+ tumor samples in MM would be useful in finding critical therapy targets as well as for making better follow-up plans. Thus, we estimated OS in the meta-analysis, taking into account that the great majority of the studies do not report this information [62].

Several published meta-analyses have attempted to evaluate the dissimilarity of tumor VM relevant to the prognosis of cancers [27, 28, 30, 42, 63]. For example, *Cao* et al. suggested that VM+ cancer patients have a poor 5-year overall survival rate compared to VM- cancer patients, particularly in metastatic diseases of sarcomas and lung, colon, liver, and melanoma cancers [19]. In contrast, *Shen* et al. addressed the tumor VM formation as an unfavorable prognostic indicator in breast cancer patients (*P* = 0.23, 95% CI: 0.08–0.38, *p = 0.003*) [64]. In line with our results, *Yang* et al. showed that tumor VM is significantly associated with cancer differentiation, lymph node metastasis and distant metastasis, (*P* = 2.16; 95% CI: 1.98–2.38; *p < 0.001*) [65]. With such foreground and assumptions, this current study allows us to reach a better understanding of the clinical role of VM formation in MM patients using statistical approaches. Conversely, the correlation between VM and survival of cancer patients remain controversial or inconclusive.

We would like to point out that there are some limitations in the current work: First, we only included papers published in English, while papers published in other languages, notably Chinese and Russian, were excluded, which certainly could cause selection bias. Also, we did not consider the sensitivity analysis when reflecting on the significant difference among individual articles. Importantly, in most selected studies, the common detection methods were IHC techniques. The different primary antibodies using a wide range of antibody dilutions might also affect the IHC sensitivity. Furthermore, the small sample size, short follow-up times, and lack of homogeneous distribution of the population (no studies dealing with the African continent) might also affect the precision of the estimates. However, the publication bias results showed that these limitations were not important enough to influence the analysis of late-stage and fatal complications. Future clinical studies with larger sample sizes, standardized protocols, and more homogeneous populations would be required to fully understand the prognostics potential of VM status of tumors in melanoma patients.

## Conclusions

The results of the present meta-analysis suggest for the first time that VM+ status of tumors is associated with a poor OS of MM patients. We also showed that VM+ status is an accurate prognostic biomarker in small sample size groups of Asian patients. Therefore, VM status could be a promising prognostic biomarker directing surgical intervention and effective adjuvant therapy of MM patients.

## Supplementary information

**Additional file 1: Table S1.** The detailed search strategy. **Table S2.** The excluded full-text articles. **Table S3.** Quality assessment of the included studies according to the Newcastle-Ottawa Scale (NOS).

**Additional file 2: Figure S1.** Risk of bias graph. The overall risk of bias was regarded as low in all qualified studies, in term of the QUADAS-2 assessment.

## Data Availability

Not applicable.

## Abbreviations

AUC: Under the curve
CAM: Comprehensive meta-analysis
CI: Confidence interval
DOR: Diagnostic odds ratio
ECM: Extracellular matrix
EKR: Extracellular signal-regulated kinas
EphA2: EPH receptor A2
HIF-1a: Hypoxia inducible factor 1a
MM: Malignant melanoma
MMP: Matrix metalloproteinase
NLR: Negative likelihood ratio
NOS: Newcastle-Ottawa scale
Notch4: Neurogenic locus notch homolog protein 4
P: Proportion
PAS: Periodic acid schiff’s
PLR: Positive likelihood ratio
PRISMA: Preferred reporting items for systematic reviews and meta-analysis
QUADAS-2: Quality assessment of diagnostic accuracy studies 2
SROC: Summary receiver operating characteristic
Twist1: Twist-related protein 1
UV: Ultraviolet
VE: Vascular endothelial
VM: Vasculogenic mimicry
