# Supplementary material for: The role of vascular mimicry as a biomarker in malignant melanoma: a systematic review and meta-analysis
Source: BMC Cancer. 2019 Nov 21;19:1134. doi: 10.1186/s12885-019-6350-5 (PMC6873453; doi:10.1186/s12885-019-6350-5)
Supplement: Supplementary file 1 — Additional file 1: Table S1. The detailed search strategy. Table S2. The excluded full-text articles. Table S3. Quality assessment of the included studies according to the Newcastle-Ottawa Scale (NOS). [file 12885_2019_6350_MOESM1_ESM.docx]

**Additional file 1**

**Title:**

**Vasculogenic Mimicry as a Poor Diagnostic and Prognostic Indicator in Patients with Malignant‎ Melanoma: A ‎Systematic Review and Meta-analysis**

**Zhenhua Zhang, Saber Imani, Marzieh Dehghan Shasaltaneh, Hossein Hosseinifard, Linglin Zou, Yu Fan, Qinglian Wen**

**Inventory of Supplemental Information**

**- Table S1‎ (Page 2-4)**

**-Table S2 (Page 5-6)**

**- Table S3 (Page 7)**

**- References (Page 8)**

# Table S1: The detailed search strategy.

| 1. **PUBMED database** |
| --- |
| **Step 1: (#1) Number: 8723**  ‎(Vascular Mimicry [MeSH Terms]) OR (Vasculogenic Mimicry) OR (Tumor Cell-lined Vessels) OR ‎‎(Tumor Derived Endothelial cells)‎  **Step 2: (#2) Number: 340,872**  (Prognosis[MeSH Terms]) OR (Survival) OR (Outcome)  **Step 3: (#3) Number: 3,928,441**  (melanoma[MeSH Terms]) OR (non-melanoma) OR (nonmelanoma) OR (basal cell carcinoma) OR (squamous cell carcinoma) OR (cancer) OR (neoplasms) OR (malignant melanoma) OR (neoplasm) OR (basal-cell skin cancer) OR (squamous-cell skin cancer) OR (Skin Neoplasms) OR (skin cancer))  **Step 4: (#1 OR #2 OR #3= #4) Number: 6,320,327**  (‎(Vascular Mimicry [MeSH Terms]) OR (Vasculogenic Mimicry) OR (Tumor Cell-lined Vessels) OR ‎‎(Tumor Derived Endothelial cells)‎ OR ‎(Prognosis[MeSH Terms]) OR (Survival) OR (Outcome) OR (melanoma[MeSH Terms]) OR (non-melanoma) OR (nonmelanoma) OR (basal cell carcinoma) OR (squamous cell carcinoma) OR (cancer) OR (neoplasms) OR (malignant melanoma) OR (neoplasm) OR (basal-cell skin cancer) OR (squamous-cell skin cancer) OR (Skin Neoplasms) OR (skin cancer))  **Step 5: (#1 And #2 = #5) Number: 3,010,459**  (‎(Vascular Mimicry [MeSH Terms]) OR (Vasculogenic Mimicry) OR (Tumor Cell-lined Vessels) OR ‎‎(Tumor Derived Endothelial cells)‎ AND ‎(Prognosis[MeSH Terms]) OR (Survival) OR (Outcome)  **Step 6: (#1 And #3 = #6) Number: 3,742,902**  (‎(Vascular Mimicry [MeSH Terms]) OR (Vasculogenic Mimicry) OR (Tumor Cell-lined Vessels) OR ‎‎(Tumor Derived Endothelial cells)‎ AND (melanoma[MeSH Terms]) OR (non-melanoma) OR (nonmelanoma) OR (basal cell carcinoma) OR (squamous cell carcinoma) OR (cancer) OR (neoplasms) OR (malignant melanoma) OR (neoplasm) OR (basal-cell skin cancer) OR (squamous-cell skin cancer) OR (Skin Neoplasms) OR (skin cancer))  **Step 7: (#2 And #3 = #7) Number: 3,645,912**  (Prognosis[MeSH Terms]) OR (Survival) OR (Outcome) AND (melanoma[MeSH Terms]) OR (non-melanoma) OR (nonmelanoma) OR (basal ‎cell carcinoma) OR (squamous cell carcinoma) OR (cancer) OR (neoplasms) OR (malignant ‎melanoma) OR (neoplasm) OR (basal-cell skin cancer) OR (squamous-cell skin cancer) OR ‎‎(Skin Neoplasms) OR (skin cancer))‎  **Step 8: (#1 And #2 And #3 = #8) Number: 238**  (‎(Vascular Mimicry [MeSH Terms]) OR (Vasculogenic Mimicry) OR (Tumor Cell-lined Vessels) OR ‎‎(Tumor Derived Endothelial cells)‎ AND ‎(Prognosis[MeSH Terms]) OR (Survival) OR (Outcome) AND (melanoma[MeSH Terms]) OR (non-melanoma) OR (nonmelanoma) OR (basal cell carcinoma) OR (squamous cell carcinoma) OR (cancer) OR (neoplasms) OR (malignant melanoma) OR (neoplasm) OR (basal-cell skin cancer) OR (squamous-cell skin cancer) OR (Skin Neoplasms) OR (skin cancer))  Timespan: All years.  Search language=Auto  **Number: 243** |
| 1. **Scopuse database** |
| (TITLE-ABS-KEY AND (melanoma) OR (non-melanoma) OR (nonmelanoma) OR (basal cell carcinoma) OR (squamous cell carcinoma) OR (cancer) OR (neoplasms) OR (malignant melanoma) OR (neoplasm) OR (basal-cell skin cancer) OR (squamous-cell skin cancer) OR (Skin Neoplasms) OR (skin cancer)) AND TITLE-ABS-KEY ("Prognosis) OR (Survival) OR (Outcome ") AND TITLE-ABS-KEY (melanoma[MeSH Terms]) OR (non-melanoma) OR (nonmelanoma) OR (basal cell carcinoma) OR (squamous cell carcinoma) OR (cancer) OR (neoplasms) OR (malignant melanoma) OR (neoplasm) OR (basal-cell skin cancer) OR (squamous-cell skin cancer) OR (Skin Neoplasms) OR (skin cancer))  **Number: 82** |
| 1. **Google Scholar database** |
| **Step 1: (#1), Number: 1,930**  ‎(Vascular Mimicry) OR (Vasculogenic Mimicry) OR (Tumor Cell-lines Vessels) ‎OR ‎‎(Tumor Derived Endothelial cells)  **Step 2: (#2), Number: 28,320**  (Prognosis) OR (Survival) OR (Outcome)  **Step 3: (#3), Number: 6,972**  (melanoma[MeSH Terms]) OR (non-melanoma) OR (nonmelanoma) OR (basal cell carcinoma) OR (squamous cell carcinoma) OR (cancer) OR (neoplasms) OR (malignant melanoma) OR (neoplasm) OR (basal-cell skin cancer) OR (squamous-cell skin cancer) OR (Skin Neoplasms) OR (skin cancer))  **Step 4: (#1 OR #2 OR #3= #4) Number: 189**  (‎(Vascular Mimicry [MeSH Terms]) OR (Vasculogenic Mimicry) OR (Tumor Cell-lined Vessels) OR ‎‎(Tumor Derived Endothelial cells)‎ OR ‎(Prognosis[MeSH Terms]) OR (Survival) OR (Outcome) OR (melanoma[MeSH Terms]) OR (non-melanoma) OR (nonmelanoma) OR (basal cell carcinoma) OR (squamous cell carcinoma) OR (cancer) OR (neoplasms) OR (malignant melanoma) OR (neoplasm) OR (basal-cell skin cancer) OR (squamous-cell skin cancer) OR (Skin Neoplasms) OR (skin cancer))  **Step 5: (#1 And #2 = #5) Number: 187**  (‎(Vascular Mimicry [MeSH Terms]) OR (Vasculogenic Mimicry) OR (Tumor Cell-lined Vessels) OR ‎‎(Tumor Derived Endothelial cells)‎ AND ‎(Prognosis[MeSH Terms]) OR (Survival) OR (Outcome)  **Step 6: (#1 And #3 = #6) Number: 29**  (‎(Vascular Mimicry [MeSH Terms]) OR (Vasculogenic Mimicry) OR (Tumor Cell-lined Vessels) OR ‎‎(Tumor Derived Endothelial cells)‎ AND (melanoma[MeSH Terms]) OR (non-melanoma) OR (nonmelanoma) OR (basal cell carcinoma) OR (squamous cell carcinoma) OR (cancer) OR (neoplasms) OR (malignant melanoma) OR (neoplasm) OR (basal-cell skin cancer) OR (squamous-cell skin cancer) OR (Skin Neoplasms) OR (skin cancer))  Timespan: All years.  Search language=Auto  **Number: 29** |
| 1. **WOS database** |
| TOPIC: (melanoma) OR (non-melanoma) OR (nonmelanoma) OR (basal cell carcinoma) OR (squamous cell carcinoma) OR (cancer) OR (neoplasms) OR (malignant melanoma) OR (neoplasm) OR (basal-cell skin cancer) OR (squamous-cell skin cancer) OR (Skin Neoplasms) OR (skin cancer)) AND TOPIC: ("Prognosis) OR (Survival) OR (Outcome ") AND TOPIC: (melanoma[MeSH Terms]) OR (non-melanoma) OR (nonmelanoma) OR (basal cell carcinoma) OR (squamous cell carcinoma) OR (cancer) OR (neoplasms) OR (malignant melanoma) OR (neoplasm) OR (basal-cell skin cancer) OR (squamous-cell skin cancer) OR (Skin Neoplasms) OR (skin cancer)  Timespan: All years.  Search language=Auto  **Number: 92** |

# Table S2: The excluded full-text articles

| **Cause 1^st^: Data without detailed information** |
| --- |
| **Number: 3**   1. Hess AR, Seftor EA, Gardner LM, Carles-Kinch K, Schneider GB, Seftor RE, et al. Molecular regulation of tumor cell vasculogenic mimicry by tyrosine phosphorylation: role of epithelial cell kinase (Eck/EphA2). Cancer research. 2001 Apr 15;61(8):3250-5. PubMed PMID: 11309274**.** 2. Pastushenko I, Vermeulen PB, Vicente-Arregui S, Van den Eynden GG, Alvarez-Alegret R, Querol I, et al. Peritumoral D2-40 Chalkley score independently predicts metastases and survival in patients with cutaneous malignant melanoma. Journal of cutaneous pathology. 2015 Oct;42(10):699-711. PubMed PMID: 26264662. 3. Ukoja V, Brandenbusch T, Tura A, Nassar K, Rohrbach DJ, Luke M, et al. [Expression of EphA2 in Metastatic and Non-Metastatic Primary Uveal Melanoma]. Klinische Monatsblatter fur Augenheilkunde. 2016 Mar;232(3):290-7. PubMed PMID: 26854480. Expression von EphA2 in metastasierten und nicht metastasierten primaren uvealen Melanomen. |
| **Cause 2^nd^: Lack of prognosis data** |
| **Number: 5**   1. Stenzel M, Tura A, Nassar K, Rohrbach JM, Grisanti S, Luke M, et al. Analysis of caveolin-1 and phosphoinositol-3 kinase expression in primary uveal melanomas. Clinical & experimental ophthalmology. 2016 Jul;44(5):400-9. PubMed PMID: 26590370. 2. Bhat P, Jakobiec FA, Folberg R. Comparison of tumor-associated vasculatures in uveal and cutaneous melanomas. Seminars in ophthalmology. 2009 May-Jun;24(3):166-71. PubMed PMID: 19437353. 3. Lederman M, Meir T, Zeschnigk M, Pe'er J, Chowers I. Inhibitor of apoptosis proteins gene expression and its correlation with prognostic factors in primary and metastatic uveal melanoma. Current eye research. 2008 Oct;33(10):876-84. PubMed PMID: 18853322. 4. Meir T, Zeschnigk M, Masshofer L, Pe'er J, Chowers I. The spatial distribution of monosomy 3 and network vasculogenic mimicry patterns in uveal melanoma. Investigative ophthalmology & visual science. 2007 May;48(5):1918-22. PubMed PMID: 17460242. 5. Coelho P, Almeida J, Prudencio C, Fernandes R, Soares R. Effect of Adipocyte Secretome in Melanoma Progression and Vasculogenic Mimicry. Journal of cellular biochemistry. 2016 Jul;117(7):1697-706. PubMed PMID: 26666522. |
| **Cause 3^th^: Without specific and clicinophtological cancer type information** |
| **Number: 5**   1. Kadkol SS, Lin AY, Barak V, Kalickman I, Leach L, Valyi-Nagy K, et al. Osteopontin expression and serum levels in metastatic uveal melanoma: a pilot study. Investigative ophthalmology & visual science. 2006 Mar;47(3):802-6. PubMed PMID: 16505010. Pubmed Central PMCID: 1414783. 2. Ziemssen F, Wegner R, Wegner J, Tatar O, Susskind D, Gelisken F, et al. Analysis of neovasculature in uveal melanoma by targeting the TGFbeta-binding receptor endoglin: is there prognostic relevance of proliferating endothelium? Graefe's archive for clinical and experimental ophthalmology = Albrecht von Graefes Archiv fur klinische und experimentelle Ophthalmologie. 2006 Sep;244(9):1124-31. PubMed PMID: 16523306. 3. Pastushenko I, Vermeulen PB, Vicente-Arregui S, Van den Eynden GG, Alvarez-Alegret R, Querol I, et al. Peritumoral D2-40 Chalkley score independently predicts metastases and survival in patients with cutaneous malignant melanoma. Journal of cutaneous pathology. 2015 Oct;42(10):699-711. PubMed PMID: 26264662. 4. Lyengar B, Singh AV. Embryonic vasculogenesis in nodular melanomas and tumour differentiation. Pathology oncology research : POR. 2011 Sep;17(3):569-77. PubMed PMID: 21203906. 5. Chang SH, Worley LA, Onken MD, Harbour JW. Prognostic biomarkers in uveal melanoma: evidence for a stem cell-like phenotype associated with metastasis. Melanoma research. 2008 Jun;18(3):191-200. PubMed PMID: 18477893. |

# Table S3: Quality assessment of the included studies according to the Newcastle-Ottawa Scale (NOS)

| **Author**  **(Ref)** | **Year** | **Case** | | **Control** | | **Comparability** | | **Exposure** | | | | **NOS score** |
| --- | --- | --- | --- | --- | --- | --- | --- | --- | --- | --- | --- | --- |
|  |  | **Definition** | **Representativeness** | **Selection** | **Definition** | **Important factors** | **Other factors** | **Secure record** | **Blind** | **Method** | **Non-response rate** |  |
| Maniotis a.j. (1) | 1999 | ★ | ★ | ★ | ★ | ★ | ☆ | ☆ | ★ | ★ | ☆ | **7** |
| Massi D. (2) | 2004 | ★ | ★ | ★ | ★ | ★ | ★ | ☆ | ☆ | ★ | ★ | **8** |
| Hillen F. (3) | 2008 | ★ | ★ | ★ | ☆ | ★ | ★ | ★ | ☆ | ★ | ★ | **8** |
| Zhang SH. (4) | 2009 | ☆ | ★ | ★ | ★ | ★ | ☆ | ★ | ☆ | ★ | ☆ | **6** |
| Shi L. (5) | 2010 | ☆ | ★ | ★ | ★ | ★ | ★ | ☆ | ☆ | ★ | ★ | **7** |
| Van Beurden V. (6) | 2012 | ☆ | ★ | ★ | ★ | ★ | ★ | ★ | ★ | ★ | ★ | **9** |
| Itzhaki O, (7) | 2013 | ★ | ★ | ★ | ☆ | ★ | ☆ | ★ | ★ | ☆ | ★ | **7** |
| Song H, (8) | 2015 | ★ | ★ | ★ | ☆ | ★ | ☆ | ★ | ☆ | ★ | ★ | **7** |
| Baocun S. (9) | 2015 | ★ | ★ | ★ | ☆ | ★ | ☆ | ★ | ★ | ★ | ★ | **8** |
| Zhao X., (10) | 2015 | ★ | ★ | ☆ | ☆ | ★ | ★ | ★ | ★ | ★ | ☆ | **7** |
| Liang X., (11) | 2017 | ☆ | ★ | ★ | ★ | ★ | ☆ | ★ | ☆ | ★ | ★ | **7** |
| Zhang W., (12) | 2017 | ☆ | ★ | ★ | ★ | ★ | ☆ | ★ | ☆ | ★ | ★ | **7** |
| **★, score value=1; ☆, score value=0;The specific item information is available from http://www.ohri.ca/programs/clinical_epidemiology/oxford.asp.** | | | | | | | | | | | | |

**References**

1. Maniotis AJ, Folberg R, Hess A, Seftor EA, Gardner LM, Pe'er J, et al. Vascular channel formation by human melanoma cells in vivo and in vitro: vasculogenic mimicry. The American journal of pathology. 1999;155(3):739-52.

2. Massi D, Franchi A, Paglierani M, Ketabchi S, Borgognoni L, Reali UM, et al. Vasculogenic mimicry has no prognostic significance in pT3 and pT4 cutaneous melanoma. Human pathology. 2004;35(4):496-502.

3. Hillen F, Baeten CI, van de Winkel A, Creytens D, van der Schaft DW, Winnepenninckx V, et al. Leukocyte infiltration and tumor cell plasticity are parameters of aggressiveness in primary cutaneous melanoma. Cancer immunology, immunotherapy : CII. 2008;57(1):97-106.

4. Zhang S, Li M, Zhang D, Xu S, Wang X, Liu Z, et al. Hypoxia influences linearly patterned programmed cell necrosis and tumor blood supply patterns formation in melanoma. Laboratory investigation; a journal of technical methods and pathology. 2009;89(5):575-86.

5. Shi L, Lei D, Ma C, Xu F, Li Y, Wang Y, et al. Clinicopathological implications of tumour-associated macrophages and vascularization in sinonasal melanoma. The Journal of international medical research. 2010;38(4):1276-86.

6. van Beurden A, Schmitz RF, van Dijk CM, Baeten CI. Periodic acid Schiff loops and blood lakes associated with metastasis in cutaneous melanoma. Melanoma research. 2012;22(6):424-9.

7. Itzhaki O, Greenberg E, Shalmon B, Kubi A, Treves AJ, Shapira-Frommer R, et al. Nicotinamide inhibits vasculogenic mimicry, an alternative vascularization pathway observed in highly aggressive melanoma. PLoS One. 2013;8(2):e57160.

8. Song H, Jing G, Wang L, Guo W, Ren G. Periodic acid-Schiff-positive loops and networks as a prognostic factor in oral mucosal melanoma. Melanoma Res. 2016;26(2):145-52.

9. Baocun S, Shiwu Z, Xiulan Z, Danfang Z, Xishan H. Pilot Study of Molecular Mechanism on Vasculogenic Mimicry in Bi-directional Differentiated Malignant Tumors. The Chinese-German Journal of Clinical Oncology. 2015;4(1):50-2.

10. Zhao X, Sun B, Li Y, Liu Y, Zhang D, Wang X, et al. Dual effects of collagenase-3 on melanoma: metastasis promotion and disruption of vasculogenic mimicry. Oncotarget. 2015;6(11):8890-9.

11. Liang X, Sun R, Zhao X, Zhang Y, Gu Q, Dong X, et al. Rictor regulates the vasculogenic mimicry of melanoma via the AKT-MMP-2/9 pathway. Journal of cellular and molecular medicine. 2017;21(12):3579-91.

12. Zhang W, Zhou P, Meng A, Zhang R, Zhou Y. Down-regulating Myoferlin inhibits the vasculogenic mimicry of melanoma via decreasing MMP-2 and inducing mesenchymal-to-epithelial transition. Journal of cellular and molecular medicine. 2017.
