# Supplementary material for: The role of vascular mimicry as a biomarker in malignant melanoma: a systematic review and meta-analysis
Source: BMC Cancer. 2019 Nov 21;19:1134. doi: 10.1186/s12885-019-6350-5 (PMC6873453; doi:10.1186/s12885-019-6350-5)

**Additional file 2**

**Title:**

**Vasculogenic Mimicry as a Poor Diagnostic and Prognostic Indicator in Patients with Malignant‎ Melanoma: A ‎Systematic Review and Meta-analysis**

**Zhenhua Zhang, Saber Imani, Marzieh Dehghan Shasaltaneh, Hossein Hosseinifard, Linglin Zou, Yu Fan, Qinglian Wen**

**Inventory of Supplemental Information**

**- Supplemental Fig. 1 (Page 2)**

**Figure S1 Risk of bias graph.** The overall risk of bias was regarded as low in all qualified studies, in term of the QUADAS-2 assessment.


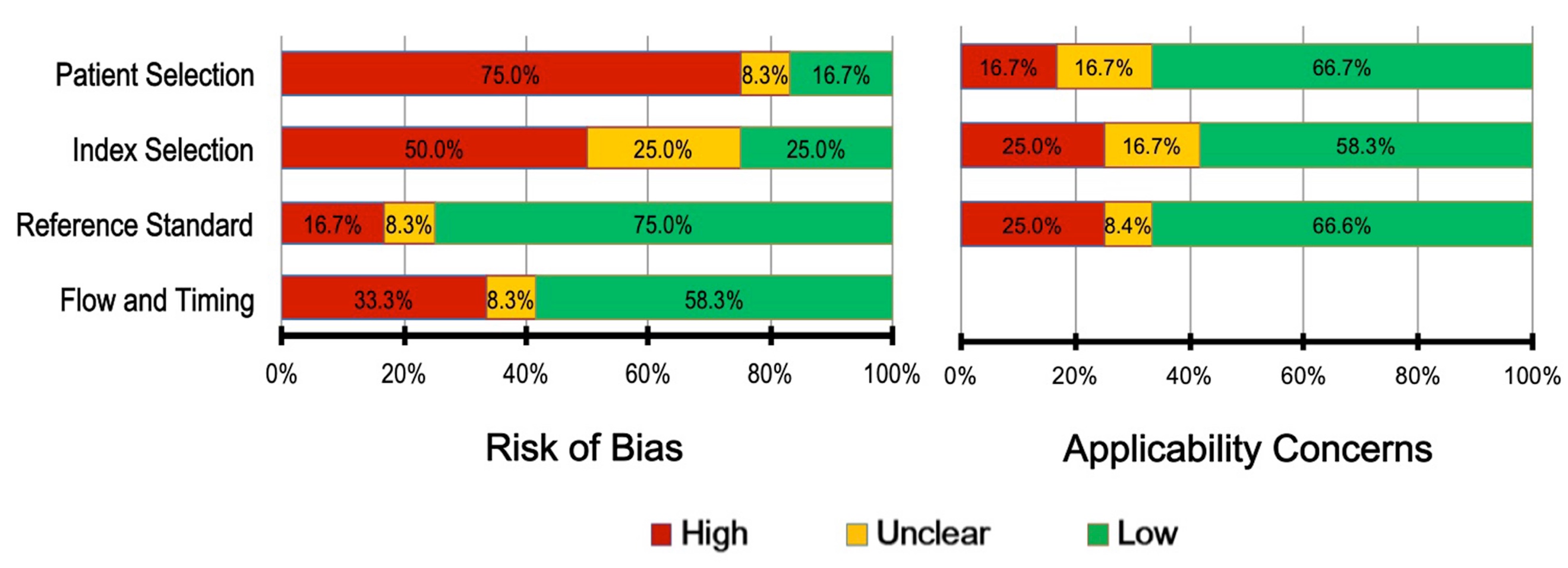

Supplement: Supplementary file 2 — Additional file 2: Figure S1. Risk of bias graph. The overall risk of bias was regarded as low in all qualified studies, in term of the QUADAS-2 assessment. [file 12885_2019_6350_MOESM2_ESM.docx]
